# Aging modifies endometrial dendritic cell function and unconventional double negative T cells in the human genital mucosa

**DOI:** 10.1186/s12979-023-00360-w

**Published:** 2023-07-14

**Authors:** Siddharth Parthasarathy, Zheng Shen, Francisco J. Carrillo-Salinas, Vidya Iyer, Alison Vogell, Diego Illanes, Charles R. Wira, Marta Rodriguez-Garcia

**Affiliations:** 1 Department of Immunology, Tufts University School of Medicine, Boston, MA USA; 2 Department of Microbiology and Immunology, Geisel School of Medicine at Dartmouth, Lebanon, NH USA; 3 Department of Gynecology and Obstetrics, Tufts Medical Center, Boston, MA USA

**Keywords:** Dendritic cells, Endometrium, Menopause, TGF-β, Sexually transmitted infections, Resident memory T cells, Double negative T cells, Single-cell sequencing

## Abstract

**Background:**

Immune function in the genital mucosa balances reproduction with protection against pathogens. As women age, genital infections, and gynecological cancer risk increase, however, the mechanisms that regulate cell-mediated immune protection in the female genital tract and how they change with aging remain poorly understood. Unconventional double negative (DN) T cells (TCRαβ + CD4-CD8-) are thought to play important roles in reproduction in mice but have yet to be characterized in the human female genital tract. Using genital tissues from women (27–77 years old), here we investigated the impact of aging on the induction, distribution, and function of DN T cells throughout the female genital tract.

**Results:**

We discovered a novel site-specific regulation of dendritic cells (DCs) and unconventional DN T cells in the genital tract that changes with age. Human genital DCs, particularly CD1a + DCs, induced proliferation of DN T cells in a TFGβ dependent manner. Importantly, induction of DN T cell proliferation, as well as specific changes in cytokine production, was enhanced in DCs from older women, indicating subset-specific regulation of DC function with increasing age. In human genital tissues, DN T cells represented a discrete T cell subset with distinct phenotypical and transcriptional profiles compared to CD4 + and CD8 + T cells. Single-cell RNA and oligo-tag antibody sequencing studies revealed that DN T cells represented a heterogeneous population with unique homeostatic, regulatory, cytotoxic, and antiviral functions. DN T cells showed relative to CD4 + and CD8 + T cells, enhanced expression of inhibitory checkpoint molecules and genes related to immune regulatory as well as innate-like anti-viral pathways. Flow cytometry analysis demonstrated that DN T cells express tissue residency markers and intracellular content of cytotoxic molecules. Interestingly, we demonstrate age-dependent and site-dependent redistribution and functional changes of genital DN T cells, with increased cytotoxic potential of endometrial DN T cells, but decreased cytotoxicity in the ectocervix as women age, with implications for reproductive failure and enhanced susceptibility to infections respectively.

**Conclusions:**

Our deep characterization of DN T cell induction and function in the female genital tract provides novel mechanistic avenues to improve reproductive outcomes, protection against infections and gynecological cancers as women age.

**Supplementary Information:**

The online version contains supplementary material available at 10.1186/s12979-023-00360-w.

## Background

The immune system in the female reproductive tract (FRT) has the double task of protecting against infections while facilitating implantation and pregnancy [1]. To accomplish this, immune cell populations in the FRT are tightly controlled by sex hormones with their distribution and functions compartmentalized in that each anatomical region displays unique immunological characteristics [1]. While most research efforts are focused on understanding the immune system in the FRT of premenopausal women as it relates to fertility and pregnancy, little is known about the immunological changes that take place in the non-pregnant endometrium in the years following menopause, when reproductive function is no longer present [2, 3].

The global population is aging rapidly, with women representing about 60% of individuals 60 and older [4]. Genitourinary infections and gynecological cancers increase with age, significantly affecting women’s morbidity and mortality [5]. Sexually transmitted infections (STI) are increasing in older women, and while the overall prevalence is lower than in younger women, the increase rate is higher, representing a public health problem [6, 7]. In women, the aging process is intimately related to menopause, when ovarian hormone production ceases marking the end of the reproductive function. Thus, it is critical to understand how the immune system changes in the FRT before and after menopause as women age. We have previously described how menopause and aging induce multiple changes in immune cell distribution and function of different immune cells in the FRT, including Th17 cells, CD8 + T cells and dendritic cells (DCs) [8–13].

DCs that reside in the mucosa provide immune surveillance against pathogens and trigger specific adaptive immune responses [14]. Unlike macrophages, DCs are specialized in priming naïve T cells and sustaining the cytokine environment during antigen presentation that shapes the immune response [15]. The tissue environment in which DCs reside strongly influences DC phenotype and function [15]. In the FRT, DCs also have the unique task of being tolerant to allogeneic antigens in order to facilitate reproductive function [3, 16]. We have previously demonstrated that different subsets of DCs exist throughout the FRT, including CD1a + DCs, that display characteristics of classical DCs, and CD14 + DCs which co-express CD11b and resemble monocyte-derived DCs [17]. Both DC subsets can induce proliferation of naïve T cells and upregulation of tissue residency markers (CD103) on naïve CD8 + T cells, suggesting their ability to control tissue resident memory T cells in the FRT [9]. Interestingly, the ability of endometrial DC to induce CD103 expression on CD8 + T cells is age-dependent and enhanced after menopause [9].

In addition to DCs, FRT tissues are populated with different T cell populations, which have distinct roles in defense during the menstrual cycle, throughout pregnancy and following menopause [1]. We have previously reported that endometrial CD8 + T cell function is strongly regulated throughout the menstrual cycle and increases after menopause [10–12]. We also demonstrated that Th17 presence increases in the EM after menopause [8]. However, little is known about other unconventional T cell subsets such as double negative TCRαβ + (DN) T cells, which are thought to play an important role in reproduction in mice [18]. The origin and function of DN T cells in humans is poorly defined, and may include multiple cells with different origins [19]. Further, in humans, DN T cells have been mostly studied in pathological conditions, such as solid tumors (melanoma), transplantation and autoimmune diseases [19–21], but little is known about tissue distribution and function in the steady state. Two recent studies described the presence of Mucosal-associated invariant T cells (MAIT) in the endometrium and cervicovaginal tissues, which represents a subset of unconventional DN T cell population with antimicrobial properties [22, 23]. However, a gap remains in our knowledge about the heterogeneity, distribution, and function of TCRαβ + DN T cells throughout the human FRT and if these cells change with aging.

Here we investigated how endometrial DCs’ ability to induce T cell proliferation is modified by aging and unexpectedly found a DN (CD4-CD8-) naïve T cell population that was being stimulated to proliferate in a DC subset-dependent and age-dependent manner. Characterization of unconventional DN T cells through single-cell sequencing and flow cytometry in human genital tissues revealed a heterogeneous tissue-resident population with unique adaptive and innate-like functions, and uncovered a selective decline of this population in the cervix with aging. Our findings provide essential information to understand immune responses related to reproductive success and defense against gynecological cancers and infections over the course of women’s lives.

## Results

### Endometrial antigen presenting cell subsets maintain differential cytokine secretion profiles during the induction of T cell proliferation

We have previously identified different subsets of antigen presenting cells in the human endometrium and demonstrated their ability to induce proliferation of naïve T cells [9, 17], a hallmark of DC function [24–26]. Here we wanted to define the cytokine secretion profile induced during the naïve T cell proliferation process to identify DC-specific functional differences between endometrial antigen presenting cell subsets.

**Fig. 1:**
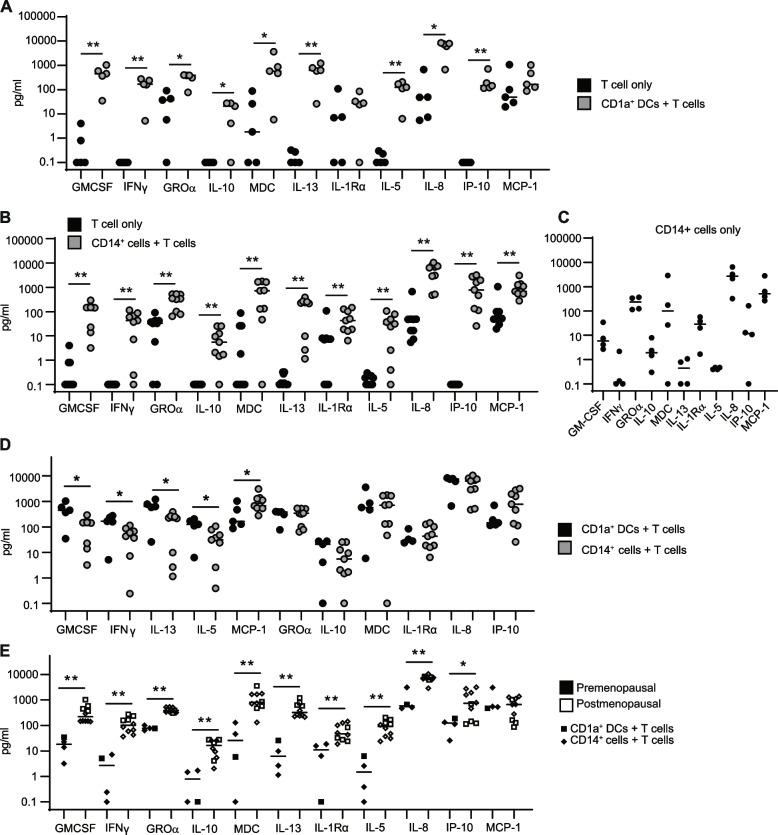
Menopause enhances cytokine profile induction by endometrial antigen presenting cells. **A** Comparison of cytokine induction in co-culture of blood naïve T cells with allogeneic endometrial CD1a + DCs and (**B**) CD14 + cells isolated from premenopausal (*n* = 4) and postmenopausal patients (*n* = 10). Black dots represent blood naïve T cells alone. **C** Production of cytokines by CD14 + cells alone ( *N* = 4). **D** Comparison of proliferating T cell cytokine induction by CD1a + (*n* = 5) and CD14 + (*n* = 9) endometrial cells in co-culture. (D) Comparison of cytokine production in co-culture of blood naïve T cells with allogeneic endometrial antigen presenting cells (CD1a + (squares) and CD14 + (diamonds)) from pre (black) and postmenopausal women (white). Mann–Whitney U test was used to compare independent groups. *p < 0.05, **p < 0.01, ***p < 0.001

We purified CD1a + and CD14 + cells from the same human endometrial samples and co-cultured them with allogeneic naïve T cells from blood as described before [9, 17, 27]. After magnetic bead selection, the CD1a + and the CD14 + purified populations expressed high levels of CD11c and HLA-DR (Supplementary Fig. 1A, Additional file 1). The CD14 + selected population contained about a 20% of cells co-expressing CD1a (Supplementary Fig. 1B, Additional file 1). Supernatants from the allogeneic co-culture were collected after 6 days to evaluate cytokine secretion in a multiplex assay as described in methods. Compared to naïve T cells alone, co-culture with CD1a + DCs resulted in a significant upregulation of most cytokines in our panel, including pro-inflammatory (GM-CSF, IFNγ), anti-inflammatory (IL-10, IL-5, IL-13) and chemokines (IL-8, GROα, MDC, IP-10) (Fig. 1A). Co-culture of CD14 + cells with naïve T cells resulted in significant upregulation of the same cytokines as CD1a + DCs and also IL1Rα and MCP-1 (Fig. 1B). IL-17a, IL-15, IL-6 and IFNα2 were undetectable (not shown). To further determine the main cell source for cytokine production, we were able to include a control with CD14 + cells alone for a subset of patients (Fig. 1C). As seen in Fig. 1B, CD14 + cells alone produced high levels of GROα, MDC, IL1Rα, IL-8, IP-10 and MCP-1, which were further upregulated in the co-culture (Fig. 1B). CD14 + cells alone did not contribute to the production of IFNγ, IL-13 or IL-5. We then compared the cytokine profiles induced during the T cell proliferation process by CD1a + and CD14 + cells. As seen in Fig. 1D, CD1a + DCs induced higher levels of GM-CSF, IFNγ, IL-13 and IL-5 compared to CD14 + cells, while CD14 + cells induced higher secretion of MCP-1, with no differences for the rest of cytokines and chemokines analyzed.

Next, we investigated if menopausal status could influence cytokine secretion profiles. For these analyses, results from CD1a + and CD14 + cells were combined to increase power, since not enough co-cultures with CD1a + DCs from premenopausal women were available for DC subset comparisons. Endometrial CD1a + and CD14 + cells from postmenopausal women induced significantly increased secretion of multiple cytokines (GM-CSF, IFNγ, GROα, IL-10, MDC, IL-13, IL1Rα, IL-5, and IL-8) during T cell proliferation compared to premenopausal women (Fig. 1E). Both CD1a + and CD14 + subsets contributed to the observed increased secretion in postmenopausal women, with the exception of IP-10, which was enhanced by CD14 + cells but not CD1a + DCs (Fig. 1E).

These findings demonstrate that cytokine secretion profiles induced by endometrial CD1a + and CD14 + cell subsets are distinct and change as women age, suggesting differential induction of T cell activation profiles.

### Endometrial DCs induce naïve T cell proliferation in a subset specific manner

Since we observed differential secretion profiles during the T cell proliferation process between CD1a + and CD14 + cells, we next investigated the extent to which these cell populations differ in their ability to induce naïve T cell proliferation, a DC-specific function [24–26]. Endometrial CD1a + and CD14 + cells from the same human endometrial samples were co-cultured with allogeneic naïve T cells from blood and proliferation evaluated by flow cytometry as described [9, 27].

**Fig. 2:**
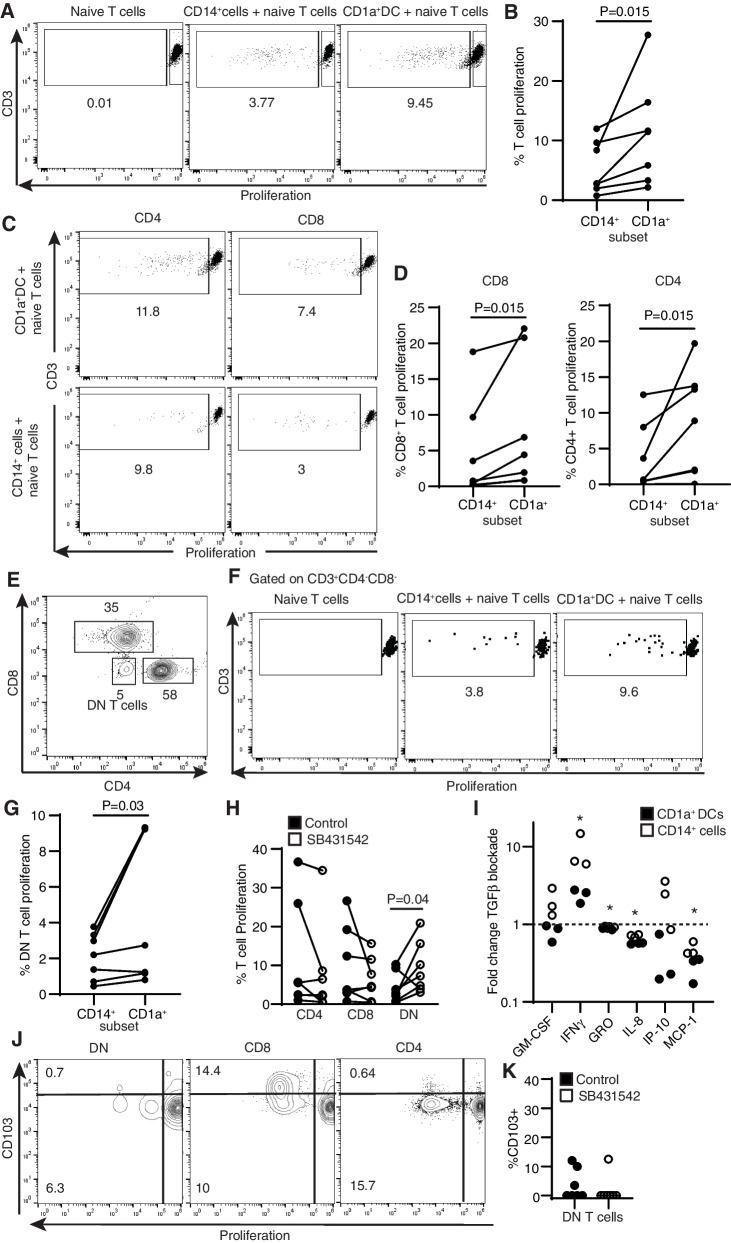
Endometrial DCs induce naïve T cell proliferation in a subset-specific manner. Allogeneic naïve T cells from blood were co-cultured with CD14 + or CD1a + cells isolated from the endometrium. **A** Representative plot demonstrating proliferation of naïve T cells in the absence and presence of CD14 + or CD1a + endometrial cells. **B** Comparison of the percentage of naïve T cell proliferation induced by CD14 + and CD1a + endometrial cells from the same women (*n* = 7). **C** Representative example of CD4 + and CD8 + naïve T cell proliferation induced by CD14 + and CD1a + endometrial antigen presenting cells. **D** Proliferation rate of CD8 + or CD4 + naïve T cells in the presence of CD14 + or CD1a + cells. **E** Flow cytometry plot of different blood naïve T cell populations after proliferation assay. **F** Representative plots demonstrating induction of DN T cell proliferation by CD14 + or CD1a + endometrial antigen presenting cells. **G** Proliferation percentage of DN T cells in response to the different endometrial antigen presenting cell subsets. Wilcoxon pair comparison was used. **H** Percentage of CD4 + , CD8 + and DN T cell proliferation in the absence (control) or presence of the TGFβ signaling inhibitor SB431542 (*n* = 7). Non-parametric Friedman test for multiple comparisons was used. **I** Fold change of cytokine production in co-culture of blood naïve T cells with allogeneic endometrial antigen presenting cells (CD1a + (black; *n* = 3) and CD14 + (white; *n* = 3)) in the presence of the TGFβ signaling inhibitor SB431542 relative to control conditions. One sample Wilcoxon signed-rank test was used to compare the median of the TGFβ blockade group to the normalized value of the controls [1]. *p < 0.05. **J** Representative plots showing expression of CD103 on DN, CD8 + and CD4 + T cells during the proliferation process induced by CD14 + or CD1a + endometrial antigen presenting cells. **K** Percentage of CD103 expression on proliferated DN T cells in the absence (control) or presence of the TGFβ signaling inhibitor SB431542 (*n* = 7)

Consistent with our previous findings, after 6 days in culture we detected naïve T cell proliferation in the presence of both CD1a + and CD14 + cells (Fig. 2A). However, CD1a + DCs induced significantly more T cell proliferation compared to CD14 + cells isolated from the same patients (Fig. 2B).

Next, we analyzed proliferation of CD4 + and CD8 + T cell populations to identify potential differences in the type of T cell subset induced. As seen in Fig. 2C, CD1a + and CD14 + cells induced proliferation of CD4 + and CD8 + T cells, but CD1a + DCs showed enhanced ability to induced proliferation of both naïve T cell populations compared to CD14 + cells (Fig. 2D). These results indicate that endometrial CD1a + DCs have enhanced ability to induce naïve T cell proliferation compared to CD14 + cells.

### Endometrial DCs induce proliferation of double negative (CD4-CD8-) T cells

During our analysis we noticed the presence of a double negative (DN) (CD4-CD8-) naïve T cell population that was also being stimulated to proliferate in addition to CD4 + and CD8 + T cells (Fig. 2E). Therefore, we examined the ability of each antigen presenting cell subset to induce DN T cell proliferation. Both CD1a + and CD14 + cells induced DN T cell proliferation (Fig. 2F), however, CD1a + DCs were superior at inducing DN T cell proliferation compared to CD14 + cells from the same patients (Fig. 2G), similar to our results with the other T cell subsets (Fig. 2D).

We have previously demonstrated that the TGFβ signaling pathway regulates genital DC ability to induce CD103 + CD8 + T cells without affecting overall proliferation induction capacity [9]. To investigate if this same pathway could be involved in induction of DN T cell proliferation by genital DCs, we blocked TGFβ signaling using the TGFβ receptor 1 blocker SB431542 during the proliferation process as described before [9]. As seen in Fig. 2H, TGFβ blockade resulted in increased proliferation of DN T cells by twofold, while no changes were detected in the proliferation of CD4 + and CD8 + T cells. To better understand the underlying mechanisms responsible for enhanced proliferation of DN T cells after TGFβ blockade, we evaluated cytokine production during the proliferation process in the presence of SB431542. TGFβ blockade significantly enhanced production of IFNγ by both CD1a + and CD14 + cells, and suppressed production of GRO, IL-8 and MCP-1 (10%, 37% and 60% reduction respectively) (Fig. 2I). GM-CSF and IP-10 followed opposite patterns by CD1a + and CD14 + cells (Fig. 2I). Next, we assessed whether proliferation of DN T cells was associated with upregulation of CD103 and if it would be modified by TGFβ. As shown in Fig. 2J, in contrast to CD8 + T cells, we detected minimal upregulation of CD103 on DN T cells during the proliferation process, with expression similar to that detected on CD4 + T cells, and TGFβ blockade did not modify CD103 expression (Fig. 2K).

Taken together, these results establish the ability of endometrial DCs to induce DN T cell proliferation through a TGFβ-dependent mechanism and demonstrate that CD1a + DCs have enhanced ability to induce naïve T cell proliferation, including CD8 + , CD4 + and DN T cell proliferation.

### Aging selectively enhances endometrial CD1a + DC ability to induce T cell proliferation

Since we observed differences in secretion profiles induced by antigen presenting cells from premenopausal and postmenopausal women, we next explored if the ability of each DC population to induce naïve T cell proliferation could be differentially affected by aging.

**Fig. 3:**
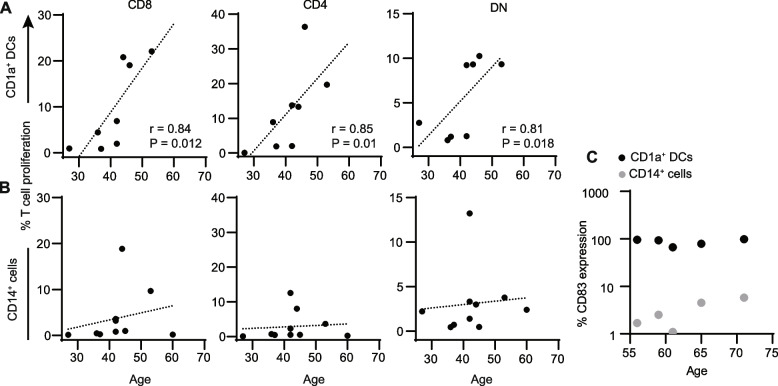
Aging selectively enhances endometrial CD1a + DC function. Correlation between age and proliferation of CD8 + , CD4 + and DN T cells induced by (**A**) CD1a + endometrial DCs (*n* = 8) and (**B**) CD14 + endometrial cells (*n* = 10). **C** Expression of CD83 by CD14 + and CD1a + endometrial cells from the same patients with respect to age of patient (*n* = 5). Spearman correlation test was used. P and r values are indicated when the correlation was significant

To identify age-dependent effects, we purified CD1a + and CD14 + cells from premenopausal and postmenopausal women, evaluated their ability to induce proliferation of naïve CD4 + , CD8 + and DN T cells, and performed correlation analyses between the age of the women from which endometrial antigen presenting cells were purified and the level of proliferation of the different T cell subsets that each antigen presenting cell population induced. As seen in Fig. 3A, with increasing age, CD1a + DCs induced increasing levels of CD8 + , CD4 + and DN T cell proliferation. In contrast, no correlation was found between age and T cell proliferation levels induced by CD14 + cells (Fig. 3B).

We have previously reported that CD1a + DCs express higher levels of CD83, a co-stimulatory molecule, than CD14 + cells [17] and therefore hypothesized that changes in CD83 expression with aging could be responsible for the age-dependent differences in induction of proliferation. However, we found no evidence of changes in the expression of CD83 in these subsets with increasing age (Fig. 3C).

These results indicate that aging selectively increases CD1a + DC ability to induce proliferation of naïve T cells, including DN T cells, whereas CD14 + cell ability to induce proliferation remains largely unchanged as women age.

### DN T cells are more abundant in the endometrium than in cervix and are redistributed with increasing age in a site-dependent manner

Recognizing that DN T cells remain undefined in human genital tissues and considering our in vitro findings that endometrial DCs induce DN T cell proliferation in an age-dependent manner, we next investigated DN T cell distribution in FRT tissues, and any potential modifications with age.

**Fig. 4:**
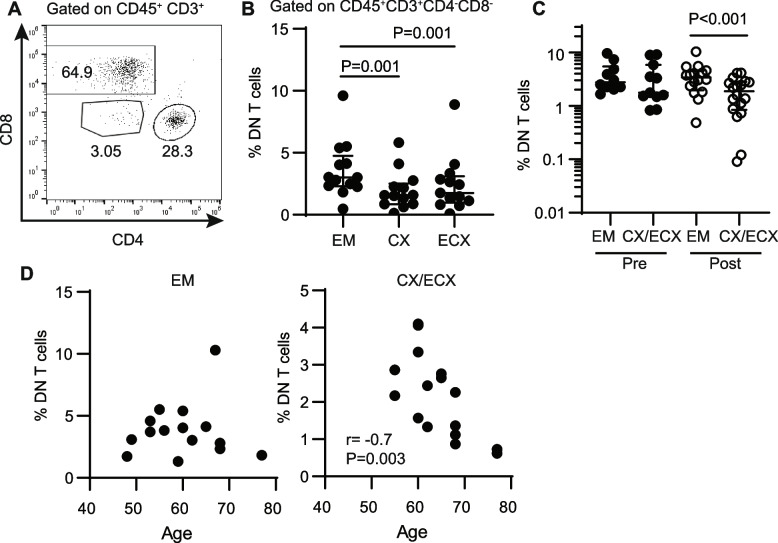
DN T cells are present in FRT tissues, are more abundant in the endometrium and show redistribution with aging in a site-dependent manner. Flow cytometry analysis of T cell populations in different anatomical regions of the FRT. **A** Representative plot of T cell populations in the FRT (EM). **B** Percentage of DN T cells in endometrium (EM), endocervix (CX) and ectocervix (ECX) from the same patients (*n* = 13; Friedman test). **C** Comparison of DN T cell percentages in EM and CX/ECX in premenopausal (pre; EM = 10, CX/ECX = 11) and postmenopausal women (post; EM = 16, CX/ECX = 19). **D** Correlation between age and percentage of DN T cells in EM (left) and CX/ECX (right) following menopause. Non-parametric paired Friedman (**B**) and unpaired Kruskal–Wallis (**C**) test followed by Dunn’s post-test. Spearman test was used in D, and P and r values are indicated when the correlation was significant

Mixed cell suspensions were generated after digestion of endometrial, endocervical and ectocervical tissues from the same patients to identify DN T cells by flow cytometry. After gating on the T cell population (CD45 + CD3 +), a DN (CD4-CD8-) T cell subset was clearly identified that represented less than 10% of the total CD3 + T cell population (Fig. 4A). DN T cells were detected in endometrium (EM), endocervix (CX) and ectocervix (ECX), but were significantly more abundant in the EM compared to the other two sites (Fig. 4B).

Next, we evaluated if the distribution of DN T cells could be affected by menopause by comparing samples obtained from premenopausal and postmenopausal women. Because no significant differences were detected in DN T cell abundance between endo and ectocervical samples (Fig. 4B), data from these two locations were combined as “cervical” for menopause and aging comparisons. As seen in Fig. 4C, when samples from premenopausal women were compared, no differences were found between DN T presence in endometrium and cervix, but, after menopause, DN T cell presence was significantly reduced in the cervix compared to the endometrium.

To evaluate if aging in the years following menopause had any additional effects on DN T cell tissue levels, we performed correlations with age in the postmenopausal group and found that DN T cells significantly declined in cervical samples but remained constant in the endometrium (Fig. 4D).

### DN T cells represent a discrete T cell subset in the FRT with distinct transcriptional profiles

Since DN T cells are poorly characterized in the human FRT, to better understand their phenotype and function, we optimized a protocol to simultaneously determine DN T cell surface expression markers and the whole transcriptome profile at the single-cell level through oligo-conjugated antibody tags and RNA sequencing.

**Fig. 5:**
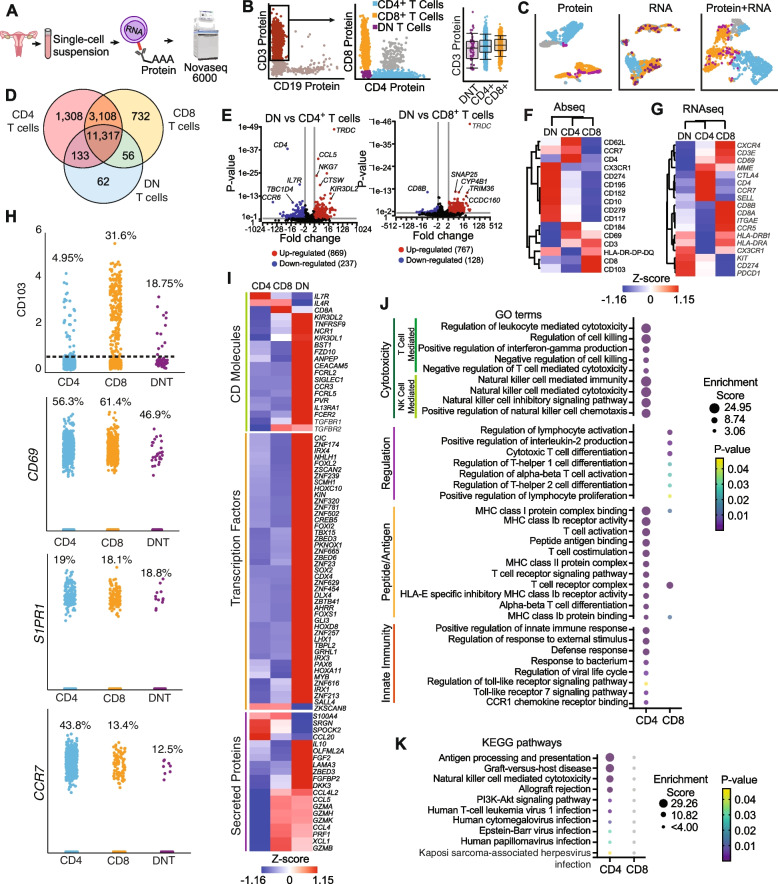
DN T cells constitute an unconventional subset of T cells in the FRT with a distinct gene expression profile. **A** Diagram of experimental procedures. **B** Identification of CD3 + DN T cells via oligo-conjugated antibody expression. **C** Analysis of protein expression, RNA expression or protein + RNA integrated data plotted in Uniform manifold approximation and projection (UMAP) graphs from T cell subsets: CD4 + (blue), CD8 + (yellow), DN (purple) and double positive T cells (grey). **D** Venn diagram shows genes expressed in DN T cells, CD4 + and CD8 + T cells. **E** Volcano plot showing differentially expressed genes comparing DN vs CD4 + T cells (left panel) and DN vs CD8 + T cells (right panel) (significance *p* ≤ 0.05; -2 < Fold Change > 2). **F** Hierarchical clustering heatmaps comparing relative expression levels of surface markers (AbSeq) and (**G**) analogous RNA expression. **H** Percentage of each T cell population expressing tissue-resident related profiles: CD103 oligo-conjugated antibody, and transcripts for CD69, S1PR1 and CCR7. **I** Heat map of selected CD molecules, transcription factors and secreted proteins differentially expressed in DN, CD4 + and CD8 + T cells. (Significance *p* ≤ 0.05; -1.2 < Fold Change > 1.2). **J** Bubble plot shows the enriched gene ontology (GO) terms significantly upregulated in DN T cells vs CD4 + or DN T cells vs CD8 + , that are associated to functions related to cytotoxicity, regulation, antigen presentation and innate immunity. **K** Bubble plot shows enriched KEGG pathways in DN T cells vs CD4 + and DN T cells vs CD8 + T cells

Mixed cell suspensions from endometrium and cervix were enriched for immune cells, incubated with oligo- conjugated antibodies and whole transcriptome profile of single cells determined by sequencing as detailed in methods (Fig. 5A). Analysis of surface proteins via oligo-conjugated antibodies identified the presence of DN T cells (CD3 + , CD4-, CD8-) (Fig. 5B, purple), reproducing our flow cytometry findings (Fig. 4A). When protein expression, gene expression profile or gene + protein integrated data were represented using uniform manifold approximation and projection (UMAP), DN T cells were identified in multiple clusters, preferentially clusters containing CD8 + T cells (Fig. 5C), suggesting heterogeneity of the DN T cell population.

To further evaluate the transcriptional differences between DN T cells, CD4 + and CD8 + T cells, we compared their whole transcriptome expression profiles. As expected, the majority of transcripts detected in DN T cells were shared with CD4 + and CD8 + T cells, although uniquely expressed genes were also identified (Fig. 5D). Analysis of differentially expressed genes (DEG) between DN T cells and CD4 + T cells identified 869 genes significantly upregulated in DN T cells, and 237 downregulated in comparison to the CD4 + T population (Fig. 5E, left panel). Similarly, comparison of DN T cells and CD8 + T cells, identified significant upregulation of 767 genes and downregulation of 128 in DN T cells (Fig. 5E, right panel), demonstrating that DN T cells are transcriptionally different from both CD4 + and CD8 + T cells.

To further characterize the phenotype of DN T cells, we analyzed the expression of surface markers included in our oligo-conjugated antibody panel. Hierarchical clustering of cell surface markers separated DN T cells from CD4 + and CD8 + T cells (Fig. 5F) and demonstrated enhanced protein surface expression of inhibitory check point molecules (PD-1, PD-L1 and CTLA-4), chemokine receptors (CX3CR1 and CCR5), molecules involved in T cell priming/activation (HLA-DR) and apoptosis (CD117 and CD10) [28, 29]. Hierarchical clustering of RNA sequencing data for these same markers also segregated DN T cells from CD4 + and CD8 + T cells (Fig. 5G) and demonstrated concomitant upregulation of protein and gene expression for some molecules, such as PD1, PDL1, CD117 (*PDCD1*, *CD274*, and *KIT*), while other markers showed opposite profiles, such as CD10 (*MME*) and CTLA4 (Fig. 5G). Intermediate expression of CD62L (*SELL*), CD69 and CD103 (*ITGAE*) in DN T cells was consistent between protein and RNA results (Fig. 5F and G). These findings highlight the importance of using integrated protein + RNA data to characterize these cells. Because CD69 and CD103 are expressed on tissue-resident T cells [30], we investigated in more detail protein/gene profiles related to tissue residency to determine if DN T cells represent a tissue resident population. Tissue resident profile can be defined by high expression of CD69 and downregulation of S1PR1 and CCR7, with CD103 expression related to residency in epithelial tissues [30]. As seen in Fig. 5H, about 20% of DN T cells expressed CD103 protein, approximately half of DN T cells expressed CD69 transcripts, less than 20% of DN T cells expressed SRP1 and around 12% expressed CCR7 transcripts, similar to CD8 + T cells. This transcriptional profile suggests the presence of tissue resident DN T cells.

To further validate our single-cell antibody findings, we performed flow cytometry staining of some of the markers identified in DN T cells and confirmed surface expression of CD10, HLA-DR, CD62L, CCR7, CX3CR1 and high expression of CCR5 (the main HIV coreceptor for mucosal infection [31]) on the DN T cell population in the different anatomical regions of the FRT (Supplementary Fig. 2, Additional file 1). Of note, CCR7 surface expression was detectable but very low (Supplementary Fig. 2E, Additional file 1), consistent with the transcriptional signature and with a tissue-resident phenotype [30]. Next, we investigated additional differentially expressed genes that encode surface markers not included in our antibody panel that could help identify and characterize DN T cells. As seen in Fig. 5I, DN T cells expressed genes that encode NK cell markers (*KIR3DL1, KIR3DL2, NCR1*), the chemokine receptor CCR3, markers involved in apoptosis (*TNFRSF9, CEACAM5*), cytokine receptors (upregulation of *IL13RA*, but downregulation of *IL4R* and *IL7R*), and markers involved in adhesion to extracellular matrix and human papillomavirus viral entry (*PVR*). We also detected expression of genes encoding TGFβ receptors (*TGFBR1* and *TGFBR2*), suggesting that DN T cells may respond to tissue derived TGFβ. Because of the NK cell related signatures and heterogeneous profile detected, we further investigated potential contribution of known T cell subsets that lack CD4 and CD8 expression, such as NKT cells, γδ T cells and MAIT cells. As shown in supplementary Fig. 3, DN T cells expressed low levels of CD56, but lacked CD16 expression, making it unlikely that NKT cells represent a major contributing subset to the DN T cell population [32] (Supplementary Fig. 3A, Additional file 1). Furthermore, DN T cells expressed less than 2% of γδTCR as determined by flow cytometry, but about 10% of DN T cells co-expressed *TRGC1/TRGC2* and *TRDC* as determined by RNA sequencing, which encode the gamma and delta chains of the γδTCR (Supplementary Fig. 3B, Additional file 1). Lastly, we detected less than 3% MR1 expression on DN T cells and by did not detect *MR1*, *SLC4A10* or *TRAV1-2* RNA expression (Supplementary Fig. 3C-D, Additional file 1), genes characteristic of MAIT cells [33]. Overall, these results suggest that these populations were not major contributing subsets to the observed signatures.

We also identified a number of transcription factors upregulated in DN T cells, relative to that seen on CD4 + and CD8 + T cells, many of which were involved in embryonic development (Fig. 5I). Of particular interest to immune and reproductive function in the FRT, we identified upregulation of transcription factors critical for ovarian development and function (*FOXL2, CREB5*), differentiation and function of female reproductive organs (*ZBED6, HOXD2, HOXA11, LHX1*), regulation of the Wtn/β-catenin pathway (*SALL4, CDX4, SOX2, ZBED3*), PPARα pathway (*AHRR, GRHL1*) and IL-4 and IL-13 signaling pathway (*SOX2*), and cellular senescence and response to DNA damage (*KIN, SCMH1*).

We also detected upregulation and downregulation of genes encoding secreted factors (Fig. 5I). Remarkably, DN T cells specifically expressed high levels of genes related to secreted molecules involved in tissue homeostasis and remodeling including basement membrane formation and function and epithelial-mesenchymal regulation (*LAMA3*), wound healing (*FGF2*) and extracellular matrix binding (*OLFML2A*). DN T cell also had upregulated expression of *CCL5* and *CCL4*, CCR5-ligands important for innate anti-HIV protection, *FGFBP2* which is secreted selectively by cytotoxic lymphocytes, *ZBED3* and *DKK3* which are related to the Wtn/β-catenin pathway and IL-10, with immunoregulatory function.

To better understand the immunological functions of DN T cells in comparison to CD4 + and CD8 + T cells we next performed gene ontology (GO) analysis of significantly upregulated genes in DN T cells. When compared to CD4 + T cells, DN T cells had significant enrichment of terms related to cytotoxicity (Fig. 5J and Supplementary Table 1, Additional file 1), including T cell mediated (T cell mediated cytotoxicity, regulation of cell killing, positive regulation of IFNγ production) and NK cell mediated cytotoxicity, terms related to MHC-I and MHC-II peptide binding and T cell signaling/activation, and terms involved in innate-like defense responses (TLR signaling, CCR1 chemokine receptor binding, response to bacterium and external stimulus), including anti-viral responses (regulation of viral life cycle, TLR7 signaling pathway) (Fig. 5J). When compared to CD8 + T cells, a smaller number of terms was significantly enriched, mostly related to regulation of immune response (regulation of IL-2 production and leukocyte/T cell activation), regulation of T cell differentiation (proliferation, Th1, Th2 and cytotoxic differentiation) and MHC-I peptide binding (Fig. 5J).

Consistently, KEGG pathway analysis, revealed enrichment of pathways related to antigen processing and presentation, graft vs host disease, NK cell mediated cytotoxicity, allograft rejection, the PI3K-akt signaling pathway (involved in cell survival and apoptosis) and several viral infection pathways, including human papillomavirus infection (HPV) (Fig. 5K). These pathways were significantly enriched in DN T cells when compared to CD4, and were present when compared to CD8 T cells, but did not reach significance (Fig. 5K).

Overall, these findings demonstrate that DN T cells are transcriptionally different than CD4 + and CD8 + T cells, and suggest that DN T cells may display functions involved in reproduction, immune regulation and defense, including anti-viral functions, through cytotoxicity and innate-like mechanisms.

### DN T cells express tissue residency markers and cytotoxic molecules

Our single-cell data analysis identified transcripts related to tissue residency and cytotoxicity in the DN T cell population. To directly determine tissue residency and cytotoxic potential of DN T cells in FRT tissues, we measured surface expression of CD69 and CD103 (markers that identify tissue-resident memory T cells and intraepithelial residency) [30, 34], and intracellular content of cytotoxic molecules by flow cytometry.

**Fig. 6:**
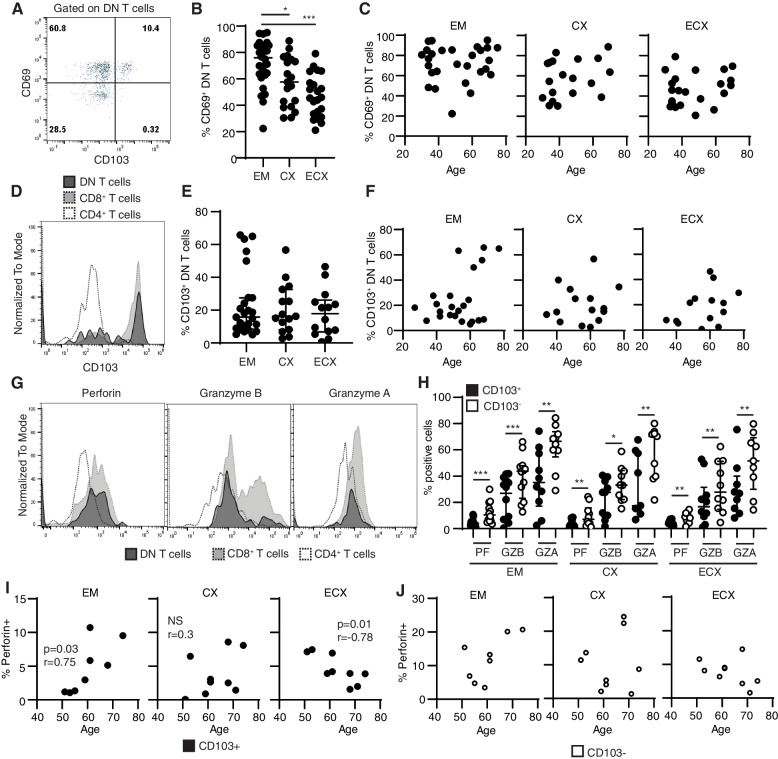
DN T cells express tissue residency markers and intracellular cytotoxic molecules. **A** Representative contour plot of CD69 and CD103 expression on DN T cells. **B** Percentage of CD69 + DN T cells in different anatomical regions of the FRT (EM = 28, CX = 19, ECX = 22). **C** Lack of correlation between CD69 + DN T cells and age in endometrium (EM = 28), endocervix (CX = 19) and ectocervix (ECX = 22). **D** Representative overlay histogram of CD103 expression by DN T cells (black), CD8 + (gray) and CD4 + (clear) T cells. **E** Percentage of CD103 + DN T cells in different anatomical regions of FRT (EM = 24, CX = 15, ECX = 14). **F** Lack of correlation between CD103 + DN T cells and age in EM (left), CX (center) and ECX (right). **G** Representative plots of Perforin (right), Granzyme B (center) and Granzyme A (left) expression by DN (black), CD8 + (gray) and CD4 + (clear) T cells in the FRT. **H** Comparison of percentage of CD103 + (black) and CD103- (white) DN T cells expressing perforin (PF), granzyme B (GZB) and granzyme A (GZA) in the endometrium (EM = 12), endocervix (CX = 10) and ectocervix (ECX = 10). Wilcoxon paired test was used to compare CD103 + and CD103- DN T cells within each tissue. *p < 0.05, **p < 0.01, ***p < 0.001. **I** Correlation between age in postmenopausal women and perforin + CD103 + DN T cells and (**J**) perforin + CD103- DN T cells in EM (*N* = 8), CX (*N* = 9) and ECX (*N* = 9). Spearman correlation analysis was performed. Each dot represents a different patient

DN T cells expressed high levels of CD69, with a subset of cells co-expressing CD103, indicating tissue-resident phenotype (Fig. 6A). Comparison of DN T cells from different anatomical compartments within the FRT (EM, CX and ECX) revealed CD69 expression on 20–95% of cells, with expression significantly higher in EM compared to CX and ECX (Fig. 6B). Next, we investigated whether aging affects CD69 expression, but no correlation was detected between the percentage of CD69 + DN T cells and age, regardless of the FRT site analyzed (Fig. 6C). DN T cells expressed CD103 at levels comparable to CD8 + T cells (Fig. 6D). DN T cells from the three FRT sites analyzed (EM, CX and ECX) expressed CD103 at equal levels, with a wide range of expression (Fig. 6E). We investigated potential effects of menopause and aging on CD103 expression, however no correlations were found (Fig. 6F).

Next, cytotoxic potential of DN T cells was evaluated by measuring intracellular content of perforin, granzyme (GZ) A and B. Intracellular staining of resting cells revealed expression of perforin, GZB and GZA, at levels intermediate in between the CD8 + and the CD4 + T cell populations (Fig. 6G). While the percentage of cells expressing perforin was similar to the CD8 + T cell population, the expression of GZB and GZA resembled the CD4 + T cell population (Fig. 6G). We have previously described that CD8 + CD103- T cells express higher levels of cytotoxic molecules compared to CD8 + CD103 + T cells [10, 11]. To determine if this differential expression was also present in the DN T cell population, we measured cytotoxic molecules in CD103 + and CD103- DN T cells. The intracellular content of perforin, GZA and GZB was significantly higher in CD103- compared to CD103 + DN T cells, and this difference was observed in the three anatomical sites analyzed (Fig. 6H). Perforin was expressed in less than 10% of CD103 + DN T cells, while the mean percent of CD103 + DN T cells expressing GZB and GZA was around 25% (Fig. 6H). In comparison, CD103- DN T cells had significantly higher expression of cytotoxic molecules, with more than double the percentage of perforin + and GZA + cells compared to CD103 + DN T cells (Fig. 6H).

Finally, we evaluated whether expression of intracellular molecules changes with aging. DN T cells expressing GZB and GZA did not change with aging (not shown), however, we observed age-dependent changes in CD103 + perforin + DN T cells in a tissue-specific manner. In the EM, perforin-expressing DN T cells increased after menopause as women aged, while a significant decline was observed in the ECX, but no effects of aging were detected in the CX (Fig. 6I). Interestingly, age-dependent changes were detected only in the tissue resident CD103 + DN T cell population (Fig. 6I), with no significant changes observed in CD103- DN T cells (Fig. 6J).

## Discussion

**Fig. 7:**
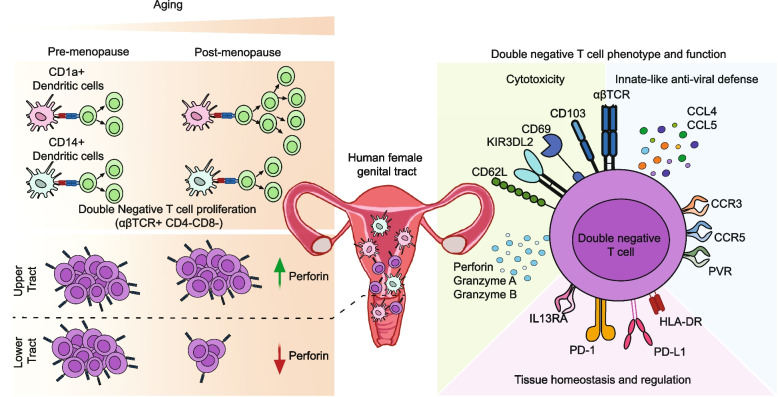
Aging regulates DN T cell induction by DCs and DN T cell distribution and function in the FRT. Schematic summary of age-dependent and DC subset specific induction of DN T cell proliferation and changes with age found in this study (top left panel); anatomical region-specific differences in DN T cell numbers with respect to aging and differences in perforin expression (bottom left panel); and phenotypic and functional characteristics of FRT DN T cells illustrating molecules associated with cytotoxicity, innate-like anti-viral defense, tissue homeostasis and regulation (right panel)

Using human genital samples from women ranging from 27 to 77 years of age, we demonstrate for the first time a compartmentalized regulation of DN T cell induction, distribution and function by aging in the female genital tract. We demonstrate that human genital DCs, particularly CD1a + DCs, induce proliferation of DN T cells, and that this function is enhanced with aging following menopause (Fig. 7, left panel). Further we demonstrate that DN T cells in genital tissues represent a functionally heterogeneous tissue-resident population with potential homeostatic, regulatory, cytotoxic, and innate-like antiviral functions and that their presence and function are selectively regulated by aging in a site-specific manner (Fig. 7, right panel). Our deep characterization of DN T cells opens new avenues for research and therapeutic potential of targeting DN T cells to improve fertility and pregnancy outcomes, gynecological cancers and antiviral protection in the female genital tract as women age.

Most studies investigating functional alterations in DCs induced by aging utilize models of in vitro-generated monocyte-derived DCs, known to be transcriptionally different than *bona fide* DCs [35, 36]. However, how aging affects different subsets of DCs in tissues is largely unknown, partly due to the technical difficulty in obtaining human tissues and purifying DC subsets. This is particularly important because tissue-resident DC subsets are conditioned and unique to the local tissue environment [15]. In this study, using primary antigen presenting cell subsets purified from the human endometrium, we demonstrate that aging and menopause regulate DC function selectively in a subset-specific manner, with CD1a + DCs being particularly sensitive to functional changes after menopause. CD1a + DCs induced DN T cell proliferation better than CD14 + cells, and this differential function between CD1a + and CD14 + cells was most pronounced as women aged due to selective enhanced proliferation induction capacity in CD1a + DCs. Unexpectedly, we found an age-dependent enhanced ability of CD1a + DCs to induce overall T proliferation (including CD4 + , CD8 + and DN T cells) when compared to CD14 + cells in our in vitro allogeneic system. This contrasted with our previous observation that no changes in DC proliferation induction capacity occurred with age [9]. These apparently disparate findings were due to an analysis in which both antigen presenting cell subsets were combined in our previous report [9], highlighting the importance to study subsets individually. Whether DN T cell induction is a specific function of CD1a + DCs or a consequence of an overall enhanced ability to induce naïve T cell proliferation remains to be determined. Enhanced ability of CD1a + DCs to induce naïve T cell proliferation is in agreement with our prior studies indicating that genital CD1a + DCs represent a homogeneous group of *bona fide* DCs, while the CD14 + population is more heterogeneous and comprises different monocyte-derived populations with phenotype and functions characteristic of DCs, including a subset of CD14 + CD1c + DCs [17].

Interestingly, although CD14 + cell ability to induce T cell proliferation remained constant after menopause, the cytokine profile induced during the proliferation process by both CD1a + and CD14 + cells changed, showing enhanced production of Th1 cytokines (IFNγ) and Th2 cytokines (IL-5, IL-10, IL-13) after menopause. Because the cytokine environment during antigen presentation is known to determine the function of induced T cells, this postmenopausal environment might support enhanced cytotoxic activity in CD8 + T cells after menopause. This would be congruent with our previous observations that cytotoxic activity of FRT CD8 + T cells is increased in postmenopausal women [10–13]. Further, our studies suggest enhanced induction of Th2 differentiation following menopause. This is in agreement with previous reports of age-dependent decline in the Th1/Th2 ratio in blood, particularly in women [37]. Of note, increased IP-10 (CXCL10) production after menopause was only detected in co-cultures with CD14 + cells. Recognizing that IP-10 has been involved in impaired T cell function and proliferation in the context of chronic viral infections [38, 39], IP-10 may be an interesting candidate molecule to explore in future studies to explain the differences in proliferation induction capacity between CD1a and CD14 DCs and changes with age detected in our study.

From a mechanistic standpoint, changes in DC proliferation induction capacity and cytokine production could be driven by the lack of hormonal control after menopause, intrinsic defects in aging DCs and the influence of the senescent tissue environment. We and others have previously shown that estradiol treatment modifies innate immune function of blood DCs [40, 41] and genital DCs [17]. We have also demonstrated that overall DC numbers decline in the FRT as women age together with tissue-resident T cells [9]. Our findings here indicate that EM CD1a + DC function is actively suppressed during the premenopausal years, most likely to prevent rejection during pregnancy, and/or that CD1a + DCs are more susceptible to intrinsic or environment-induced aging effects. Future studies to investigate the involvement of sex hormones in regulation of CD1a + DC function in the FRT are warranted.

Another novel contribution of our study is the deep phenotypic, molecular and functional characterization of DN T cells in the FRT. We demonstrate that DN T cells are present throughout the human FRT and that they are more abundant in the endometrium, which may be consistent with their previously reported involvement in reproduction in animal models [18]. The phenotype and function of DN T cells in humans remains unclear due to the low frequency of these cells and the lack of standardized definitions, particularly as it relates to human tissues. Here, using oligo-conjugated antibody tags and RNA single-cell sequencing in combination with flow cytometry, we describe for the first time that genital DN T cells represent a transcriptionally distinct and functionally heterogeneous population of unconventional T cells with a broad spectrum of potential functions, including cytotoxicity, T cell regulation, antigen presentation and innate-like functions. Our transcriptional identification of functionally heterogeneous DN subsets is consistent with a recent characterization of DN T cells in mouse spleen using single-cell sequencing and the identification of T helper, cytotoxic and innate immune functions [42]. These shared transcriptional signatures between our study and prior studies may represent common defining profiles for DN T cells. In addition, we identified potentially unique characteristics of genital DN T cells that may be relevant for homeostatic functions in the genital tract (such us maintenance of barrier function and reproductive function) and the potential involvement of DN T cells in genital viral infections, such as HPV and HIV infection. Involvement in mucosal HIV infection is further supported by a previous report describing DN T cell presence at the endocervical mucosal surface of female sex workers [43]. These unique signatures may be driven by the tissue environment as described previously for tissue-resident memory CD8 + T cells [30], a topic that deserves further investigation. We report that a large proportion of DN T cells display a tissue-resident phenotype (CD69, CD103 and low CCR7) [30, 34], and that the majority of DN T cells display an effector memory or effector cell phenotype (CD62L-CCR7-), consistent with what has been described for other T cell subsets in the FRT [2, 31, 44]. Further, we identify a number of markers with regulatory functions, including KIR3DL, inhibitory checkpoint molecules, HLA-DR, as well as cytokine and chemokine receptors and secreted factors that can be further researched for potential therapeutic purposes. Interestingly, in contrast to previous reports with mice DN T cells [42], we did not detect a Th17 signature in our samples, which could be unique to the FRT and consistent with the reported negative effect of Th17 cells in pregnancy outcomes [45]. Another unique finding in our human genital samples was that DN T cells expressed higher levels of *IL13Rα1* when compared to CD4 + and CD8 + T cells, instead of *IL7R* as described in splenic mouse DN T cells [42]. IL13 receptor α1 dimerizes with IL4 receptor and mediates signaling by IL13 and IL4. Importantly, IL13 signaling has been implicated in implantation and embryo tolerance [46, 47], supporting specific roles of DN T cells in reproduction as described by others [18, 48].

Regarding cytotoxic potential of DN T cells, we demonstrate that genital DN T cells express low levels of perforin, similar to those found in FRT CD8 + T cells [10, 11], and lower levels of GZA and GZB than FRT CD8 + T cells [10, 11], which overall would suggest low cytotoxic potential under resting conditions. However, granzymes are also known to have other immunoregulatory functions, particularly GZA has inflammatory properties [49, 50] and therefore further studies are needed to define the role of perforin and granzyme positive DN T cells in the FRT.

Recent reports have described the presence of DN MAIT cells in the EM and cervix [22, 23], which play important roles in antimicrobial control. However, MAIT cells minimally contributed to the DN T cell signatures in our single-cell preparations, or in flow cytometry analysis of MR1. The reason for this discrepancy remains to be address and may be due to the rare nature of these cells in human genital tissues. Future studies are needed to determine tissue distribution and functional differences between αβTCR + DN T cells and DN MAIT cells in the human FRT.

Finally, we uncovered site-specific effects of aging on DN T cell distribution and function. While we observed no changes in endometrial DN T cells, we detected a significant reduction in CX and ECX from postmenopausal women as they aged. We have previously described a reduction in the proportion of tissue-resident CD103 + CD8 + T cells in the cervix with no changes in the EM [9]. In contrast, in the present study we did not observe any changes in the proportion of tissue-resident CD103 + DN T cell population with aging, suggesting a selective decline of CD103 + CD8 + T cells with aging, but not of all CD103 + expressing T cells. This age-dependent differential control may be physiologically relevant since CD103 allows interactions with epithelial cells, and CD103 + CD8 + T cells represent the majority of human intraepithelial lymphocytes [51]. Our results suggest that as women age declining numbers of CD8 + intraepithelial lymphocytes may be replaced by DN CD103 + T cells. Further, we detected specific modifications in the proportion of perforin-expressing CD103 + DN T cells with aging in a tissue-specific manner. Reduction of DN T cell presence in the lower FRT, and reduced production of perforin by ectocervical DN T cells, could represent a novel factor to explain increased inflammation and reduced protection against genital infections in women as they age. Future studies including microscopy are needed to address changes in intraepithelial lymphocyte composition with aging and their potential functional consequences.

In contrast to the ectocervix, enhanced perforin production by endometrial CD103 + DN T cells may represent a mechanism of sustained endometrial barrier defense in older women, potentially triggered by lack of sex hormones, decreased CD8 + T cell presence, epithelial alterations and microbiome changes with aging [2]. While the mechanisms involved in the site-specific decrease of selected T cell populations between the EM and cervix with age as well as the functional consequences remain to be explored, it is tempting to speculate that enhanced function of endometrial CD1a + DCs observed in our study may contribute to the maintenance of defined T cell subsets in the EM. Studies are needed to determine additional functional changes in DN T cells with aging as well as the interplay between CD8 + T cells and DN T cells as women age and their role in reproduction and immune protection.

Finally, limitations of our study need to be taken into consideration for data interpretation. Our sample size is limited and additional studies with larger populations are needed to validate and expand our results. Particularly, our single-cell sequencing data needs to be confirmed and expanded with multiple women throughout their lifespan. The reduced number of DN T cells obtained from our hysterectomy samples, given their rare nature, limits the number of comparisons that can be made and the study of DN T cell subsets contributing to the functional heterogeneity of this population. Lastly, we only evaluated DC function with the allogeneic reaction assay due to lack of access to matching naïve T cells from blood or lymph nodes from the same women, and therefore autologous proliferation assays remain to be tested in future studies to fully characterize DC functional changes with age.

## Conclusions

In conclusion, we demonstrate that endometrial CD1a + DCs induce more potent T cell responses than CD14 + cells, including the induction of DN T cells. Further, aging and menopausal status selectively modify endometrial DC proliferation-induction capacity and increase cytokine/chemokine production, with CD1a + cells more susceptible to regulation after menopause. DN T cells present throughout the FRT, represent a transcriptionally unique population of unconventional T cells with broad adaptive and innate-like functions and are specifically reduced in the cervix as women age. Understanding how aging and menopausal status modify endometrial DC function as well as induction of T cell responses and regulation of T cell populations in the FRT will help tailor preventive and therapeutic strategies to improve pregnancy outcomes, gynecological cancers and genital infections for all women as they age.

## Materials and methods

### Study design

Studies were approved by Dartmouth College Institutional Review Board and the Committee for the Protection of Human Subjects (CPHS) and by the Health Sciences Institutional Review Board at Tufts University. Written informed consent was obtained before surgery from HIV-negative women undergoing hysterectomies at Dartmouth-Hitchcock Medical Center (Lebanon, NH) or at Tufts Medical Center (TMC, Boston, MA). Patients with gynecological cancer were excluded from the study. Surgery was performed to treat benign conditions including fibroids, prolapse, dysmenorrhea and abnormal uterine bleeding. For a subset of patients, information about specific surgical indication was not available. Trained pathologists selected tissue samples from endometrium (EM), endocervix (CX) and ectocervix (ECX) free of pathological lesions and distant from the sites of pathology. Women were HIV- and HPV- but no additional information regarding other genital infections was available. A total of 94 women were included in the study, ranging from 27 to 77 years of age (median = 55).

### Tissue processing

Tissues from the EM, CX and ECX were obtained after hysterectomy. In some cases, only endometrial tissue was provided by pathology. Vaginal tissues were not available. Tissues were processed to obtain a stromal cell suspension as described previously [9, 17, 27], using 0.05% collagenase type IV (Sigma-Aldrich, St. Louis, MO) and 0.01% DNAse (Worthington Biochemical, Lakewood, NJ). After filtering through a 20 µm mesh screen (Small Parts) to separate epithelial cells from stromal cells, stromal cells underwent dead cell removal (Dead Cell Removal Kit, Miltenyi biotech, Auburn, CA) as previously described [8]. This protocol results in more than 90% cell viability by trypan blue staining. After dead cell removal, mixed cell suspensions were used for phenotypical analyses by flow cytometry, or further processed for cell isolation.

### CD14 + and CD1a + cell isolation

Mixed cell suspensions were centrifuged by standard Ficoll gradient as described previously [9, 17, 27], prior to DC isolation using positive magnetic bead selection with either the CD14 + or CD1a + isolation kits (Miltenyi Biotec) according to the manufacturer’s instructions. After two rounds of positive selection, purity of the CD14 + and CD1a + population was about 90% [9, 17, 27]. Isolated DCs were plated in round bottom ultra-low attachment 96-well plates (Corning, Corning, NY) in Xvivo15 media (Invitrogen Waltham, MA) supplemented with 10% human AB serum (Valley Biomedical Winchester, VA) for in vitro allogeneic stimulation.

### Allogeneic naïve T cell stimulation assay

Naïve T cells were purified from cryopreserved peripheral blood mononuclear cells (PBMCs) using the naïve Pan T Cell Isolation Kit (Miltenyi Biotec, Auburn, CA). After purification, isolated naïve T cells were > 99% CCR7 + CD45A + as previously described [9]. Naïve T cells were stained with Cell Proliferation Dye eFluor-670 (eBioscience, San Diego, CA) as recommended by the manufacturer. Purified mucosal CD1a + or CD14 + cells (5 × 10^3^ cells) were plated with naïve T cells (7.5 × 10^4^ cells) (1:15 ratio) in round-bottom 96-well plates, in Xvivo 15 media (Invitrogen, Waltham, MA) supplemented with 10% human AB serum (Innovative Research, Novi, MI). DC to T cell ratio was selected based on our prior studies [9, 17, 27] and in vivo animal models [52]. Naïve T cells were isolated from four different blood donors and each donor was used in co-culture with DCs from at least two patients. After 6 days in culture, proliferation of T cells was assessed by flow cytometry after staining with zombie yellow dye (Biolegend, San Diego, CA) and CD3-APC-Cy7, CD8-FITC (Tonbo, San Diego, CA), CD4-PE, CD103-PE-Cy7 (eBioscience, San Diego, CA) and CD11c-PerCp-Cy5.5 (Biolegend**,** San Diego, CA**).** Naïve T cells alone were used as a negative control. For a subset of experiments, TGFβ Receptor 1 blocker, SB431542 (10 µM, Tocris Cookson Inc) was added to the media at the beginning of each co-culture as described before [9].

### Cytokine secretion determination

After 6 days in culture, supernatants from the DC-T cell cocultures were collected and a panel of 13 cytokines was detected using Luminex Assay (GM-CSF, IFNγ, GROα, IL-10, MDC, IL-13, IL-15, IL-17A, IL-1Rα, IL-5, IL-8, IP-10 and MCP-1). Undetectable values were assigned a value of 0.1 to allow for presentation in log scale.

### Flow cytometry

Mixed cell suspensions were stained for surface markers with combinations of the following antibodies: CD45-vioblue450, CD8-FITC, CD19-APC (Tonbo, San Diego, CA), HLA-DR-FITC, CD3-viogreen, CD103-PE (Miltenyi Biotec, Auburn, CA**),** CD11c-PerCp-Cy5.5, CD103-PE-Cy7, CD4-PE (eBioscience, San Diego, CA), CD56-APC (BD Pharmingen, San Diego, CA), CD69-BrilliantViolet510 (BioLegend, San Diego, CA)**.** Dead cells were excluded with 7AAD (Southern Biotech, Birmingham, AL) or zombie dye yellow staining (BioLegend, San Diego, CA). For spectral flow cytometry, the following antibodies were used: CX3CR1-PE eFluor610 (Thermo Fisher, Waltham, MA), CD3-VioGreen (Miltenyi Biotec), CD4-BrilliantUV805, CD10-BrilliantViolet650, CCR5-PE-Cy5, CD45-BrilliantUV395 (BD Biosciences, Franklin Lakes, NJ), HLA-DR-AlexaFluor700, CCR7-BrilliantViolet750, CD8-SparkBlue550, CD62L-BrilliantViolet605 (BioLegend). Analysis was performed on Gallios (Beckman Coulter, Brea, CA) or Cytek Aurora 5 lasers configuration (Cytek, Fremont, CA) flow cytometers and data analyzed with FlowJo **(**Tree Star, Inc., Ashland, OR) or OMIQ software (www.omiq.ai). Expression of surface markers is shown as percentage of positive cells. Fluorescence minus one (FMO) strategy was used to establish appropriate gates.

### Intracellular staining of cytotoxic molecules

Detection of perforin, GZA and GZB was performed on mixed cell populations after dead cell removal as described [10, 11]. Cells were surface stained first and then fixed and permeabilized with Cytofix/cytoperm kit (BD Bioscience, Franklin Lakes, NJ) according to instructions. Intracellular staining of perforin, Granzyme A and B were done using combinations of the following antibodies: anti-human Perforin-PE/Dazzle, Granzyme A-AF647, Granzyme A-PerCp-Cy5.5, Granzyme B-AF647 (BioLegend, San Diego, CA) and Granzyme B-BV421 (BD Bioscience, Franklin Lakes, NJ) as described [10, 11]. Analysis was performed on BioRad ZE5 flow cytometers (BioRad) using Everest software and data analyzed with FlowJo software (Tree Star, Inc. Ashland, OR). Expression of surface markers was measured by the percentage of positive cells. Fluorescence minus one (FMO) strategy was used to establish appropriate gates.

### Sample preparation for single-cell antibody and RNA sequencing

Single cell antibody and RNA sequencing was performed using the BD Rhapsody platform (BD Biosciences). Hysterectomy tissues (endometrium and ectocervix) from one patient were minced and enzymatically digested with Tumor Dissociation Kit (Mitenyi Biotec) and dissociated in a gentleMACS Octo Dissociator (Miltenyi Biotec) followed by sequential filtering to obtain mixed single-cell suspensions. Mixed cell suspensions from EM and ECX were incubated with oligo-conjugated antibodies and barcoded sample tags to differentiate between the two samples. 20 min post incubation, cells were washed to remove unbound protein and barcoded sample tags. Cells were counted and 10,000 cells from each tissue combined and loaded onto a BD Rhapsody™ cartridge. RNA-capture beads containing unique molecular identifier (UMI) barcodes were subsequently loaded into the cartridge followed by a lysis step to release cellular RNA. Sequencing libraries are then generated as instructed by the manufacturer.

### Sequencing and data processing

A total of 20,000 cells were sequenced in an S4 cell of NovaSeq6000 to generate raw FASTQ files with 100 base pair read length. Sequencing parameters were calculated to generate 60,000 reads per cell for whole transcriptome analysis, 600 reads per cell for sample tag barcoding and 850 reads per oligo-conjugated antibody per cell. FASTQ files were subsequently trimmed to 75 base pair read length, aligned, and annotated with the human genome to obtain gene counts by using manufacturer provided analysis pipeline. Gene counts were then uploaded to Partek® Flow® software, v10.0 for further analysis. Data was cleaned up to filter out cells expressing higher than 30% mitochondrial genes, less than 200 and greater than 4000 features per cell. Data matrix was split to separate out RNA and protein data and subsequently normalized. To enrich for immune cells, we selected cells expressing *PTPRC* (CD45) gene followed by generating principal components. Thereafter we selected cells expressing CD3 protein to enrich for T Cells (Fig. 5B). To visualize our cells of interest, we performed principal component analysis followed by generating UMAP plots. To integrate protein expression and RNA expression data, we performed weighted-nearest neighbor analysis tool on the software. To evaluate transcriptional differences between the T cell subsets, we performed differential expression analysis using ANOVA and identified uniquely upregulated and downregulated genes (*p* < 0.05; -1.2 < Log_2_(FoldChange) > 1.2). Subsequently we generated hierarchical-clustering heat maps by calculating the mean expression of genes across the different subsets. We curated lists of genes for specific categories to generate hierarchical clustering heatmaps. To generate a list of gene ontology (GO) processes, we used gene lists of upregulated and downregulated genes mentioned above and considered significant processes with *p* ≤ 0.05. To detect uniquely upregulated pathways, we used the upregulated gene list for comparing with the KEGG database and considered significant pathways with *p* ≤ 0.05. Bubble plots were generated using GraphPad Prism v9.0.

### Statistics

Data analysis was performed using the GraphPad Prism 8.0 software. A two-sided *P*-value < 0.05 was considered statistically significant. Comparison of two groups was performed with the non-parametric Mann Whitney U test or Wilcoxon paired test. Comparison of three or more groups was performed applying the non-parametric Kruskal–Wallis or paired Friedman test followed by Dunn’s post-test. Correlation analyses were performed applying non-parametric Spearman test. Power and sample size calculations were performed using anticipated means based on our prior publications [9, 10]. Data are represented as the median ± interquartile range.

## Supplementary Information

**Additional file 1: Supplementary Fig. 1.** Cell phenotype after magnetic bead selection. (A) Representative flow cytometry dot plots of CD11c and HLA-DR expression, before selection (no selection) and after CD1a+ and CD14+ DC selection.  (B) Representative flow cytometry dot plots of CD1a expression and overlay histogram of CD1a expression following CD1a+ and CD14+ DC selection. **Supplementary Fig. 2.** Phenotype of DN T cells in the FRT. Ectocervical, endocervical and endometrial representative flow cytometry plots and percentage of DN T cells expressing CX3CR1+ (A), CD10+ (B), CCR5+ (C), HLA-DR+ (D), CD62L+ CCR7- (E) and CD62L- CCR7+ (F). Each dot represents a different patient. Wilcoxon test was used for statistical analysis.  *p<0.05. ECX: ectocervix; CX: endocervix; EM: endometrium; DN: double negative. **Supplementary Fig. 3.** Characterization of CD3+CD4-CD8- T cell subpopulations. (A) Determination of NKT cell presence. Representative flow cytometry plots of CD56 and CD16 expression in CD8+, CD4+, DNT and NK cells in the FRT, and KLRB1 gene expression across T cell subsets in the FRT.  (B) Determination of γδ T cells. Representative flow cytometry plot assessing expression of γδTCR chain in DNT cells from the FRT (FMO=fluorescence minus one) and scatter plot comparing T cell subsets in the FRT for expression of TRGC2 and TRDC genes. Numbers indicate the percentage of T cells co-expressing TRGC2 and TRDC genes within each population. (C) Representative flow cytometry plots and quantification of MR1 expression on T cell populations. (D) Percentage of T cell populations expressing canonical MAIT cell genes MR1 (left), SLC4A10 (center) and TRAV1-2 (right). **Supplementary Table 1.** GO Biological Processes enhanced in DNT cells compared to CD4 T Cells.

## Data Availability

All data necessary to evaluate the conclusions in the paper are available in the main text or the supplementary materials. The single-cell sequencing data was deposited in NCBI GEO.”
