# Supplementary material for: Aging modifies endometrial dendritic cell function and unconventional double negative T cells in the human genital mucosa
Source: Immun Ageing. 2023 Jul 14;20:34. doi: 10.1186/s12979-023-00360-w (PMC10347869; doi:10.1186/s12979-023-00360-w)
Supplement: Supplementary file 1 — Additional file 1: Supplementary Fig. 1. Cell phenotype after magnetic bead selection. (A) Representative flow cytometry dot plots of CD11c and HLA-DR expression, before selection (no selection) and after CD1a+ and CD14+ DC selection. (B) Representative flow cytometry dot plots of CD1a expression and overlay histogram of CD1a expression following CD1a+ and CD14+ DC selection. Supplementary Fig. 2. Phenotype of DN T cells in the FRT. Ectocervical, endocervical and endometrial representative flow cytometry plots and percentage of DN T cells expressing CX3CR1+ (A), CD10+ (B), CCR5+ (C), HLA-DR+ (D), CD62L+ CCR7- (E) and CD62L- CCR7+ (F). Each dot represents a different patient. Wilcoxon test was used for statistical analysis. *p<0.05. ECX: ectocervix; CX: endocervix; EM: endometrium; DN: double negative. Supplementary Fig. 3. Characterization of CD3+CD4-CD8- T cell subpopulations. (A) Determination of NKT cell presence. Representative flow cytometry plots of CD56 and CD16 expression in CD8+, CD4+, DNT and NK cells in the FRT, and KLRB1 gene expression across T cell subsets in the FRT. (B) Determination of γδ T cells. Representative flow cytometry plot assessing expression of γδTCR chain in DNT cells from the FRT (FMO=fluorescence minus one) and scatter plot comparing T cell subsets in the FRT for expression of TRGC2 and TRDC genes. Numbers indicate the percentage of T cells co-expressing TRGC2 and TRDC genes within each population. (C) Representative flow cytometry plots and quantification of MR1 expression on T cell populations. (D) Percentage of T cell populations expressing canonical MAIT cell genes MR1 (left), SLC4A10 (center) and TRAV1-2 (right). Supplementary Table 1. GO Biological Processes enhanced in DNT cells compared to CD4 T Cells. [file 12979_2023_360_MOESM1_ESM.pdf]

Additional File 1

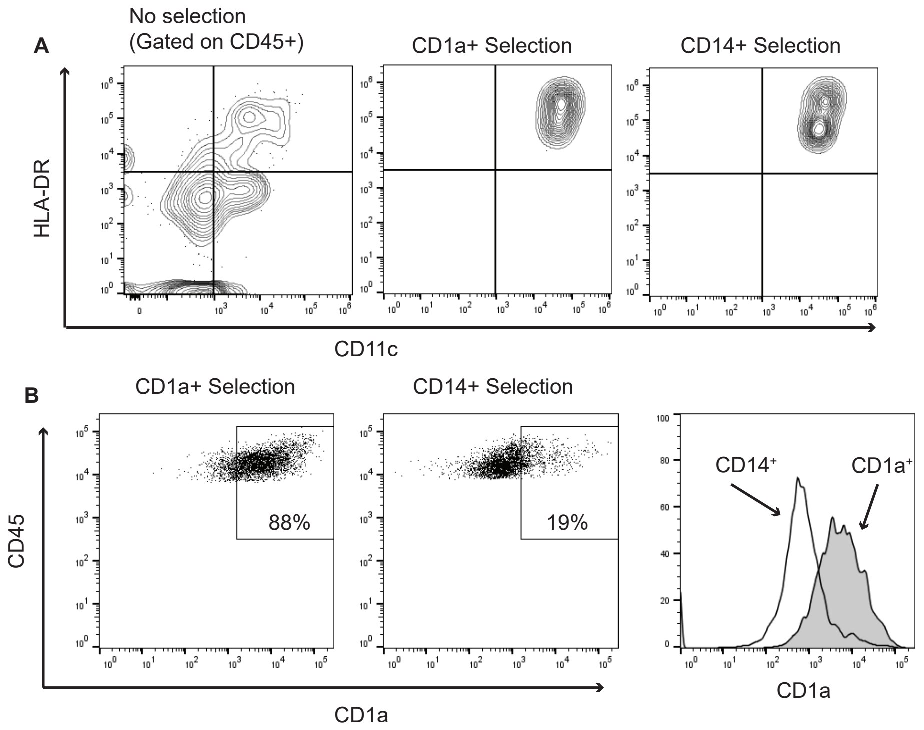

Supplementary Fig. 1. Cell phenotype after magnetic bead selection. (A) Representative flow cytometry contour plots of CD11c and HLA-DR expression, before selection (no selection) and after CD1a+ and CD14+ DC selection. (B) Representative flow cytometry dot plots of CD1a expression and overlay histogram of CD1a expression following CD1a+ and CD14+ DC selection.

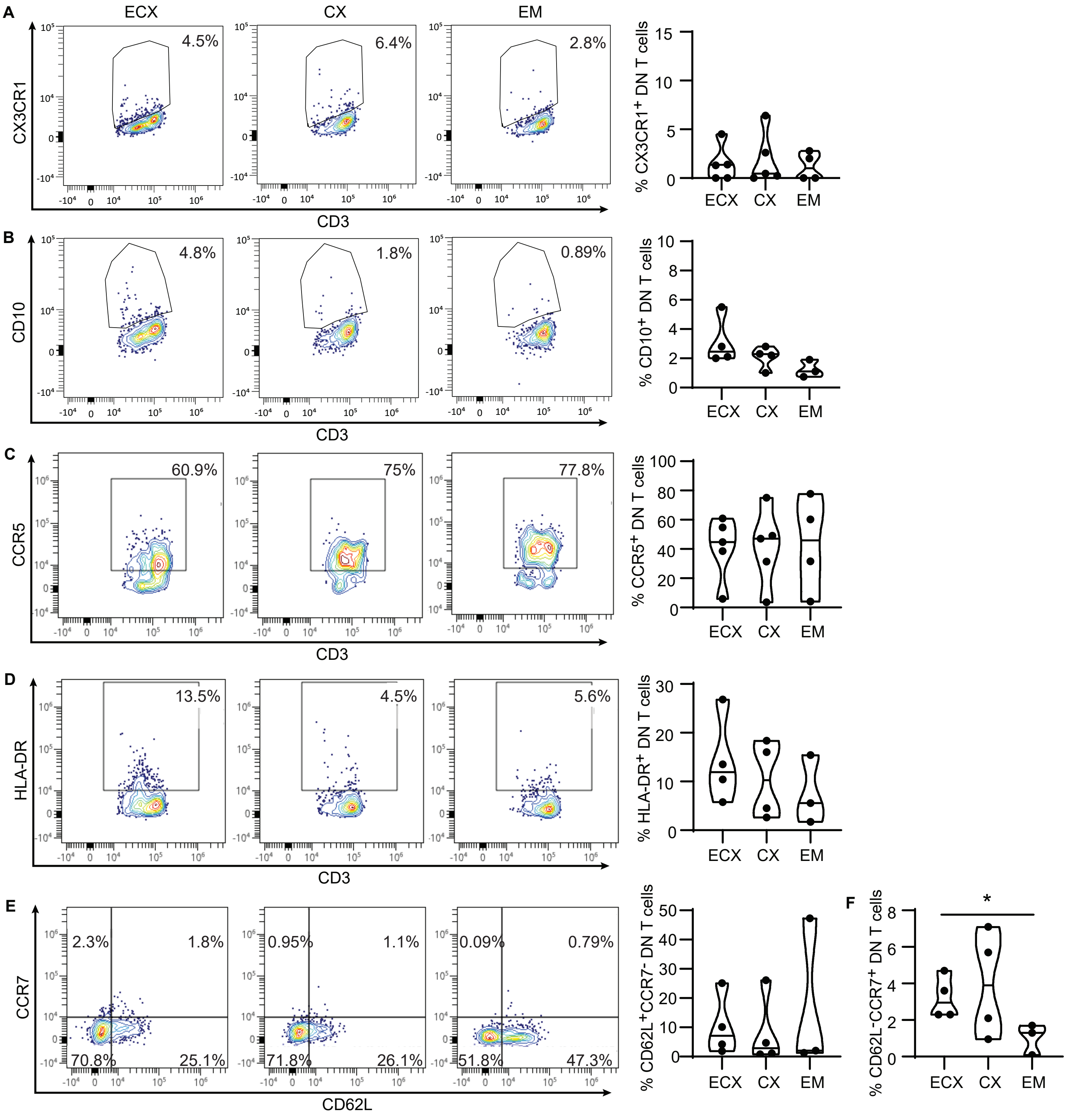

Supplementary Fig. 2. Phenotype of DN T cells in the FRT. Ectocervical, endocervical and endometrial representative flow cytometry plots and percentage of DN T cells expressing CX3CR1+ (A), CD10+ (B), CCR5+ (C), HLA-DR+ (D), CD62L+ CCR7- (E) and CD62L- CCR7+ (F). Each dot represents a different patient. Wilcoxon test was used for statistical analysis. *p<0.05. ECX: ectocervix; CX: endocervix; EM: endometrium; DN: double negative.

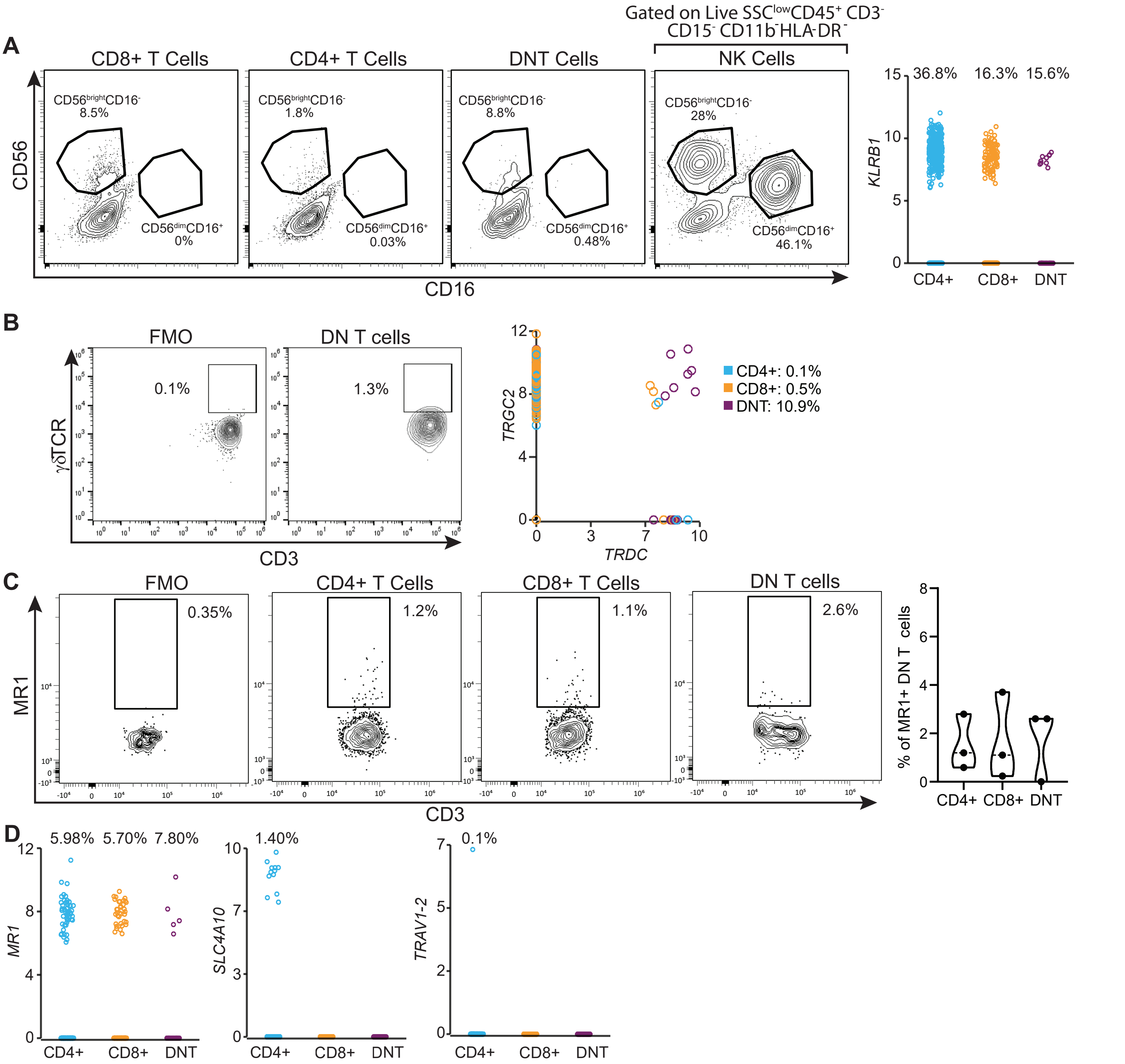

Supplementary Fig. 3. Characterization of CD3+CD4-CD8- T cell subpopulations. (A) Determination of NKT cell presence. Representative flow cytometry plots of CD56 and CD16 expression in CD8+, CD4+, DNT and NK cells in the FRT, and KLRB1 gene expression across T cell subsets in the FRT. (B) Determination of γδ T cells. Representative flow cytometry plot assessing expression of γδTCR chain in DNT cells from the FRT (FMO=fluorescence minus one) and scatter plot comparing T cell subsets in the FRT for expression of TRGC2 and TRDC genes. Numbers indicate the percentage of T cells co-expressing TRGC2 and TRDC genes within each population. (C) Representative flow cytometry plots and quantification of MR1 expression on T cell populations. (D) Percentage of T cell populations expressing canonical MAIT cell genes MR1 (left), SLC4A10 (center) and TRAV1-2 (right).

Table S1. GO Biological Processes enhanced in DNT cells compared to CD4 T Cells

Category	Name	Enrichment Score	p-Value
NK Mediated	regulation of natural killer cell mediated cytotoxicity	22.0627	2.62E-10
NK Mediated	regulation of natural killer cell mediated immunity	21.0211	7.42E-10
NK Mediated	positive regulation of natural killer cell mediated immunity	13.803	1.01E-06
NK Mediated	negative regulation of natural killer cell mediated cytotoxicity	11.101	1.51E-05
NK Mediated	negative regulation of natural killer cell mediated immunity	10.8109	2.02E-05
NK Mediated	regulation of natural killer cell mediated immune response to tumor cell	7.61809	0.00049
NK Mediated	natural killer cell mediated cytotoxicity	7.32743	0.00066
NK Mediated	natural killer cell inhibitory signaling pathway	6.88794	0.00102
NK Mediated	positive regulation of natural killer cell chemotaxis	5.70869	0.00332
Peptide Binding	MHC protein complex binding	18.3495	1.07E-08
Peptide Binding	MHC class I protein complex binding	18.181	1.27E-08
Peptide Binding	MHC class Ib receptor activity	18.181	1.27E-08
Peptide Binding	MHC protein binding	15.1963	2.51E-07
Peptide Binding	T cell activation	15.155	2.62E-07
Peptide Binding	antigen binding	13.8958	9.23E-07
Peptide Binding	peptide antigen binding	13.3322	1.62E-06
Peptide Binding	T cell costimulation	12.2414	4.83E-06
Peptide Binding	regulation of antigen receptor-mediated signaling pathway	10.5478	2.63E-05
Peptide Binding	MHC protein complex	10.0393	4.36E-05

Figure S1. **Phenotype of DN T cells in the FRT**. Ectocervical, endocervical and endometrial representative flow cytometry plots and percentage of DN T cells expressing CX3CR1+ **(A)**, CD10+ **(B)**, CCR5+ **(C),** HLA-DR+ **(D)**, CD62L+ CCR7- **(E)** and CD62L- CCR7+ **(F)**. Each dot represents a different patient. Wilcoxon test was used for statistical analysis. ECX: ectocervix; CX: endocervix; EM: endometrium; DN: double negative.

Peptide Binding	MHC class II protein complex	9.52066	7.33E-05
Peptide Binding	T cell receptor signaling pathway	9.28912	9.24E-05
Peptide Binding	MHC class II protein binding	9.01159	0.00012
Peptide Binding	T cell receptor complex	8.68153	0.00017
Peptide Binding	signaling receptor binding	8.49227	0.00021
Peptide Binding	MHC class I receptor activity	8.46585	0.00021
Peptide Binding	inhibitory MHC class Ib receptor activity	7.97416	0.00034
Peptide Binding	HLA-E specific inhibitory MHC class Ib receptor activity	7.97416	0.00034
Peptide Binding	alpha-beta T cell activation	7.95635	0.00035
Peptide Binding	CD8-positive, alpha-beta T cell activation	7.61809	0.00049
Peptide Binding	alpha-beta T cell differentiation	7.21318	0.00074
Peptide Binding	regulation of T cell mediated immunity	7.16962	0.00077
Peptide Binding	alpha-beta T cell activation involved in immune response	7.15102	0.00078
Peptide Binding	alpha-beta T cell differentiation involved in immune response	7.15102	0.00078
Peptide Binding	CD8-positive, alpha-beta T cell differentiation	5.70869	0.00332
Peptide Binding	MHC class II protein complex binding	5.63713	0.00356
Peptide Binding	antigen processing and presentation of endogenous peptide antigen	5.63713	0.00356
Peptide Binding	MHC class Ib protein binding	5.31556	0.00491
Cytotoxicity	regulation of leukocyte mediated cytotoxicity	24.9463	1.47E-11
Cytotoxicity	regulation of cell killing	23.7505	4.84E-11
Cytotoxicity	regulation of leukocyte mediated immunity	20.6039	1.13E-09
Cytotoxicity	positive regulation of cell killing	18.9934	5.64E-09
Cytotoxicity	positive regulation of leukocyte mediated cytotoxicity	17.0048	4.12E-08
Cytotoxicity	regulation of interferon-gamma production	14.0559	7.86E-07
Cytotoxicity	positive regulation of interferon-gamma production	12.0761	5.69E-06
Cytotoxicity	negative regulation of cell killing	11.4643	1.05E-05
Cytotoxicity	leukocyte mediated cytotoxicity	9.33079	8.87E-05

Cytotoxicity	negative regulation of T cell mediated cytotoxicity	7.27525	0.00069
Cytotoxicity	negative regulation of T cell mediated cytotoxicity	7.27525	0.00069
Cytotoxicity	granzyme-mediated programmed cell death signaling pathway	9.01159	1.22E-04
Innate-defense	positive regulation of innate immune response	11.1758	1.40E-05
Innate-defense	regulation of response to external stimulus	10.6777	2.31E-05
Innate-defense	defense response	10.6593	2.35E-05
Innate-defense	regulation of innate immune response	10.6162	2.45E-05
Innate-defense	response to bacterium	6.24736	1.94E-03
Innate-defense	CCR1 chemokine receptor binding	5.70869	3.32E-03
Innate-defense	toll-like receptor 7 signaling pathway	5.70869	3.32E-03
Innate-defense	regulation of viral life cycle	5.49914	4.69E-03
Innate-defense	ovarian cumulus expansion	5.36306	1.02E-03
